# A cone opsin double knockout mouse reveals long-term cone survival and provides a platform for gene therapy

**DOI:** 10.21203/rs.3.rs-9768355/v1

**Published:** 2026-06-17

**Authors:** Mikayla L. Puska, Michelle M. Giarmarco, James A. Kuchenbecker, Yajun Tang, Jose Rojas, Maureen Neitz, Jay Neitz

**Affiliations:** University of Washington; University of Washington; University of Washington; Regeneron Pharmaceuticals; Regeneron Pharmaceuticals; University of Washington; University of Washington

## Abstract

Mutations in cone opsin-encoding genes are associated with colorblindness and retinal disease. In certain forms of blue cone monochromacy (BCM) in humans, a viable cone population that does not express cone opsin is present in the retina. Double cone opsin knockout (DKO) mouse models of BCM have been developed previously using gene trap insertion in one or both opsin genes. Here, we report and characterize a new mouse line that completely lacks both short-wavelength (S) and middle-wavelength (M) sensitive cone opsin genes but maintains a viable cone population to one year old. DKO mice experienced approximately a 30% reduction in cones between 4 and 12 months, similar to the age-related decline in wild-type controls, and did not show signs of retinal degeneration. This mouse will be useful for the development of cone opsin gene therapies directed at treating BCM, in which the expression of L and M opsin is lost, but many cones remain as potential gene therapy targets. It also has implications for understanding the survival of cones without opsin, which differs from the fate of rods without rhodopsin.

## Introduction

Cone photoreceptors mediate daylight and color vision by expressing visual pigments in their outer segments. Most humans express three cone opsins, S (short), M (middle), and L (long) wavelength-sensitive, which support trichromatic color vision.^1,2,3^ Red-green color vision deficiencies result from the loss of L or M cone opsin expression and are among the most common inherited visual disorders.^4,5^ More severe defects in cone opsin expression can produce blue cone monochromacy (BCM), a rare X-linked disorder in which functional L and M cone opsin expression is absent.^6^ Although BCM patients retain S-cone function, over 90% of cones in the human retina normally express L or M opsin^7,8^ and affected individuals exhibit markedly reduced cone-mediated vision.

Importantly, studies of BCM indicate that, depending on the causative mutation, a substantial population of cones lacking functional opsin can remain viable, making these cells potential targets for gene replacement therapy. However, the long-term survival and structural integrity of opsin-less cones remain incompletely understood. Previous studies of opsin deletions in mice have produced differing results. Deletion of rhodopsin causes rapid retinal degeneration,^9^ whereas cone opsin knockout models have shown variable phenotypes. Ma et al. described a double cone opsin knockout mouse generated using gene-trap insertions that exhibited significant retinal degeneration by three months of age.^10^ In contrast, Xu et al. reported a different double cone opsin knockout model with long-term cone survival and minimal degeneration.^11^ Notably, these previously published models retained hypomorphic S-opsin alleles rather than the complete deletion of both cone opsin loci.

Here, we describe and characterize a new cone opsin double knockout (DKO) mouse generated in collaboration with Regeneron in which both cone opsin genes, including all coding exons and proximal regulatory elements, were completely removed using gene editing approaches. Mice normally express only S and M cone opsins, and a large fraction of mouse cones co-express both pigments. Consequently, deletion of both cone opsins produces a retina containing viable cones that lack cone opsin expression entirely, analogous to the opsin-less cones observed in certain forms of human BCM. This makes the model particularly well-suited for evaluating therapeutic strategies aimed at restoring cone function through gene replacement.

To assess the effects of complete cone opsin deletion, DKO mice were compared with wild-type (WT) controls, an M-opsin knockout (MKO) line generated using the same strategy, and the retinal degeneration model AIPL1-/−. We show that cones remain viable in DKO retinas for at least one year despite the complete absence of cone opsin expression, supporting the use of this model as a platform for studies of cone survival and gene therapy for BCM.

## Results

Single guide RNAs (gRNAs) were designed to target and collapse the Opn1mw and Opn1sw genes (Table 1). Mouse embryonic stem cells (hybrid C57BL/6NTac:129S6SvEvTac F1 background in which the Crb1 rd8 mutation was corrected) were genetically modified by co-electroporating gene-specific gRNAs plus SpCas9-encoded plasmid, and candidate clones were transiently selected using 1.5 ug/mL puromycin. Gene collapses in mES cells were confirmed by Taqman qPCR as previously described.^12^

**Figure 1:**
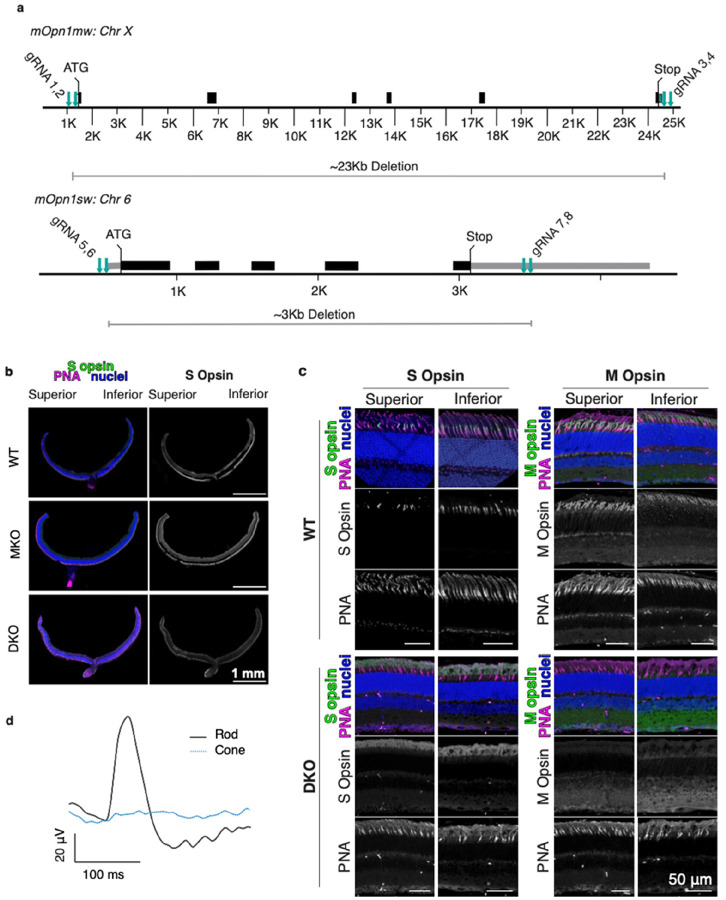
DKO retinas do not express cone opsins. **a)** Opn1mw and Opn1sw CRISPR/Cas9 assisted KO strategy. **b)** Confocal images showing central retinal sections from 4 m/o animals stained for S opsin and cone inner-outer segment sheaths (PNA); nuclei were counterstained with Hoechst. **c)** Confocal images showing inferior and superior regions of 4 m/o retina stained for S opsin (left) or M opsin (right) and PNA. **d)** Electroretinogram recordings from DKO retina under mesopic (rod-mediated) and photopic (cone-mediated) conditions.

To confirm the absence of opsin expression in the DKO model, retinal sections were stained with immunohistochemistry against cone opsins, combined with stains for cone inner-outer segment sheaths and nuclei. In whole retina images, S opsin expression was seen on the inferior portions of the MKO and WT retinas (Fig. 1b) but absent in DKO retina except for a small amount of visible autofluorescence. S and M opsins were present in WT retina, with increased M expression in the superior retina and increased S expression in the inferior retina (Fig. 1c), as is typical in wild-type mice.^3^ DKO retina lacked both S and M opsin in all regions.

ERG data recorded from DKO mice showed rod activity in scotopic and mesopic lighting conditions and no cone activity in the photopic lighting conditions (Fig. 1d). This demonstrates the presence of a healthy rod population that is able to respond to stimuli, and no response from the opsin-less cone population.

**Figure 2:**
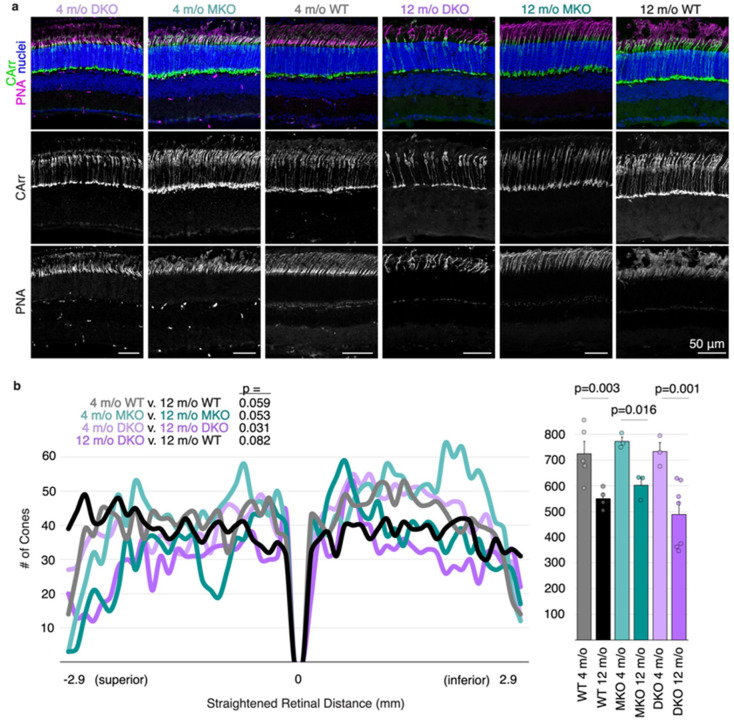
DKO retinas have a population of healthy cones. **a)** Confocal images showing the superior regions of WT, MKO, and DKO retina stained for cone arrestin (CArr) and the cone outer segment sheath (PNA). **b)** Quantification of cone numbers across whole, straightened retinal sections, displayed as a histogram of cone numbers per 100 μm bin moving superior to inferior (left), or as average total cone populations (right). P values were calculated using two–sample Kolmogorov–Smirnov tests to compare cone distributions, or Mann-Whitney tests to compare means.

To assess cone health, retina sections were stained for cone arrestin and the cone inner-outer segment sheath. Cone populations in 4 m/o WT, DKO, and MKO animals exhibited similar density and overall morphology (Fig. 2A). 12 m/o WT, DKO, and MKO animals have reduced cone populations, which we quantified using whole central retina sections from multiple animals. Cone number decreased significantly between 4 m/o and 12 m/o in all three strains (Fig. 2B; 32% ± 8% for WT, 33% ± 5% for DKO, 28% ± 3% for MKO); these changes were most prominent in the retinal periphery and are likely due to age.^13^ At both time points, the average cone population in both knockout models was similar to WT mice, suggesting the DKO and MKO retinas maintain a healthy and robust cone population.

Interestingly, MKO retina showed a subtle difference in cone loss between the inferior (opsin-expressing) and superior (opsin-less) regions. Between 4 m/o and 12 m/o, cone populations in the superior retina dropped 37% ± 7% (p = 0.01), but only 23% ± 8% in the inferior retina (p = 0.06). By contrast, cone loss between inferior and superior regions of WT and DKO retinas was similar. This trend suggests that opsin deletion may slightly accelerate decreases in cone number associated with age.

**Figure 3:**
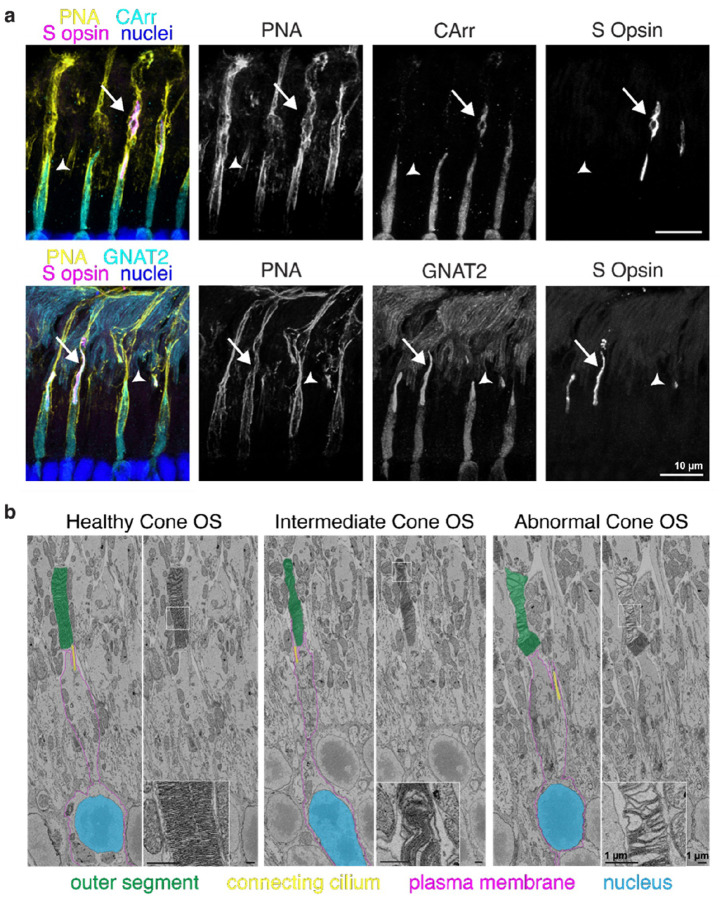
Opsin loss may disturb outer segment ultrastructure in cones. **a)** Confocal images showing cone outer segments in MKO superior retina. Sections counterstained for cone arrestin (CArr) or cone transducin (GNAT2). S opsin expressing cones (arrows) are intermixed with opsin-less cones (arrowheads). **b)** Electron microscopy images showing cone outer segments in MKO inferior retina. Presented cells are less than 20 μm away from each other, and display a mixed population of healthy, intermediate, and disorganized cone outer segments.

High-resolution confocal imaging of cones labelled for outer segment proteins revealed a mixed population of healthy, S opsin expressing cones with orderly outer segments, and opsin-less cones with truncated outer segments (Fig. 3A). Opsin-less cones trafficked the phototransduction proteins transducin and arrestin (Fig. 3A and 3B, arrowheads) to outer segments, but trafficking to outer segment tips was impaired compared to neighboring S opsin expressing cones (arrows).

We next used serial block-face scanning electron microscopy (SBFSEM) to investigate outer segment ultrastructure of MKO cones in the inferior retina. Cones were identified based on nuclear and mitochondrial architecture, and all were found to make a connecting cilium and outer segment. A mixed population of cone outer segments was seen (Fig. 3C), similar to the light microscopy findings. Many cones had neatly stacked outer segment lamellae, while neighboring cones displayed disorganized outer segments.

**Figure 4:**
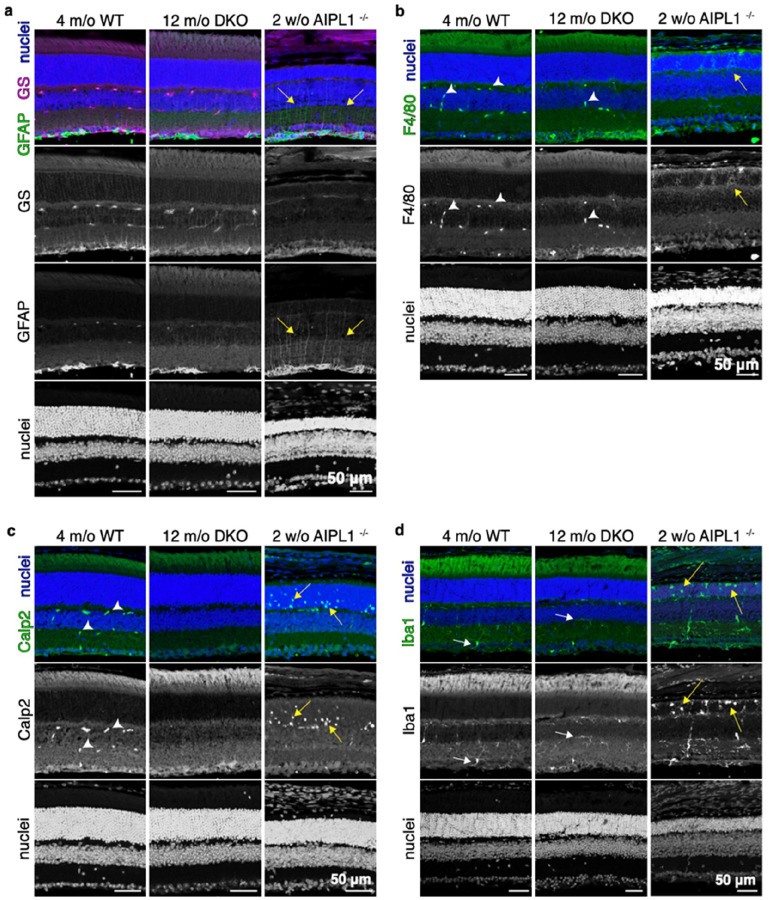
DKO retinas do not show signs of retinal degeneration at 12 m/o. Confocal images of 4 m/o WT, 12 m/o DKO, and 2 w/o AIPL1^−/−^ retinas stained for degeneration markers. **a)** Muller glia activation visualized using antibodies against glutamine synthase (GS) to label all Muller glia, and glial fibrillary acidic protein (GFAP) to label activated Muller glia. Yellow arrows – activated Muller glia in the AIPL1^−/−^ model. **b)** Macrophage recruitment visualized using an antibody against F4/80. Yellow arrow – recruited macrophages in the AIPL1^−/−^ model outer retina layer, white arrowheads – blood vessel autofluorescence. **c)** Non-apoptotic dying cells visualized using an antibody against Calpain-2 (Calp2). Yellow arrows – nuclei of dying photoreceptors in the outer nuclear layer in the AIPL1^−/−^ retina, white arrowheads – blood vessel autofluorescence. **d)** Microglia activation visualized using an antibody against Iba1. Yellow arrows – activated microglia in the photoreceptor layer of the AIPL1^−/−^ model, white arrows – homeostatic inner retina microglia.

Reduced cone populations at 12 m/o could be a sign of slow retinal degeneration, where loss of neurons sends the retina into an inflamed state. To explore this possibility, we stained both retinal sections and whole-mounts for the presence of four degeneration markers. In addition to staining DKO sections, we stained WT and AIPL1^−/−^ sections as controls; the AIPL ^−/−^ sections were in a state of active degeneration and were used to validate antibodies. No areas of degeneration were found across whole-mount retinas of DKO or MKO animals (data not shown, n = 2 animals each); images of sections from 4 m/o WT, 12 m/o DKO, and 2 w/o control AIPL1^−/−^ retina sections are presented in Fig. 4:

1. Muller glia activation has been reported in many retinal diseases, and mass activation can be detrimental to the health of the retina.^14^ Expression of the activation marker GFAP was observed in the degenerating AIPL1^−/−^ retina. By contrast, Müller glia activation was not seen in any DKO or WT samples in any region of the retina (Fig. 4A).
2. Macrophage recruitment is a sign of an active immune response in the retina.^15^ The AIPL1^−/−^ sample showed the presence of recruited macrophages expressing F4/80, indicating that the retinal degeneration had triggered an immune response. This was not apparent in the DKO or WT retinas across multiple animals (Fig. 4B).
3. Typically, when photoreceptors die during retinal degeneration, markers of non-apoptotic cell death are expressed.^16^ When staining for the presence of non-apoptotic dying cells in the photoreceptor layers using the marker Calpain-2, nuclei of dying photoreceptor cells were seen in the outer nuclear layer of the AIPL1^−/−^ sample, but not WT and DKO retinas (Fig. 4C).
4. Microglia typically assist in maintaining normal function in the retina and the brain. Microglia reactivity, which is triggered by chemical messengers released by dying photoreceptors, is a protective response to widespread photoreceptor death but can be a driving force in retinal degeneration when it leads to inflammation in the retina.^17^ Staining for microglia activation using the marker Iba1 showed a large microglia response in the AIPL1^−/−^ photoreceptor layer, but only homeostatic inner retina microglia were seen in the WT and DKO sections (Fig. 4D).

Together, these experiments demonstrate that this cone opsin DKO mouse model fully lacks both cone opsins but maintains a robust population of cones. DKO cone numbers decrease between 4 m/o and 12 m/o, similar to aged WT, but the retina is not in an active state of degeneration.

## Discussion

We characterized the first mouse line having undergone complete deletion of both cone opsin genes. Immunostaining and ERG recordings confirmed the absence of cone opsin expression and function in the DKO retinas (Fig. 1), supporting the efficacy of the gene editing methods used by Regeneron. This DKO mouse model maintains a healthy population of cones up to 12 m/o despite the lack of S and M opsin expression. Over a year, cone numbers decrease around 30% in DKO retina, comparable to the age-related decline seen in WT retina (Fig. 2). Despite reduced cone numbers and disorganized cone outer segments (Fig. 3), 12 m/o DKO retinas did not show signs of active retinal degeneration (Fig. 4).

In previous research on opsin deletions in mice, Lem et al. found that deleting rhodopsin leads to rapid retinal degeneration, beginning with the rods and extending to many other cells in the retina.^9^ In more recent research on the effects of cone opsin gene disruption, Ma et al. characterized a double cone opsin knockout mouse model in 2022. Their mouse model was developed by crossing a mouse line with a Neomycin resistance cassette inserted into the S opsin gene between the third and fourth exon^18^ with a mouse line with an M opsin gene trap insertion.^19^ This model experienced significant cone degeneration by three months old.^10^ The same year, Xu et al. published research on the retina of a different double cone opsin knockout mouse model that did not experience retinal degeneration. Theirs was generated by breeding the same S opsin knockout line as Ma's group and a mouse line with a CRISPR gene deletion of the M opsin gene.^20^ The resulting DKO mice did not experience significant cone degeneration for at least 12 months.^11^

Our DKO mice do not express any portion of either cone opsin gene (Fig. 1), and the presence of a healthy cone population reinforces findings from Xu et al. that cone opsin is not integral for cone viability.^11^ This contrasts with the loss of rhodopsin in rods, which leads to severe retinal degeneration.^9^ Rods and cones exhibit physical differences in outer and inner segment structure, as well as differences in the compositions of their lipid membranes.^21^ Additionally, the composition of the outer segment sheaths in the extracellular matrix microenvironment of the rods and cones is different.^22^ The different outer sheaths mediate interactions with the RPE for each of these cell types.^23^ Cones also rely on the Rod Derived Cone Viability Factor (RdCVF), a protein secreted from rods that promotes cone metabolism.^24^ Rods do not rely on metabolic support from the cone population, so alterations to cones are less likely to impact rods. Further, the mouse retina is 97% rod-dominated,^25^ and loss of such a large cell volume in the photoreceptor layer could disrupt the physical environment for surviving cones. These differences in outer segment architecture, metabolic relationships, and relative cell numbers could play a role in the survival of rods with and without viable cone populations.

In C57BL/6J mouse retinas, 30% of rods are lost, and cone numbers decline between 3 and 12 months.^13^ Additionally, both cone and rod electroretinogram (ERG) responses decrease over time,^26^ suggestive of cone loss with age in WT mice. We observed approximately a 30% decrease in the total cone population in WT and knockout retinas between 4 m/o and 12 m/o (Fig. 2). Opsin-less cones may be more susceptible to age-related changes, but a majority of cones remain in the first year.

Considerations about retinal architecture in the absence of cone opsins are important for any future gene therapy treatments. Gene therapies aim to restore light sensitivity to cones with missing opsins; for this to effectively mediate vision, cones must correctly signal to interneurons in the inner retina, including horizontal, bipolar, amacrine, and ganglion cells.

In rhodopsin mutation models, ERG responses in rods and cones differ from WT responses even prior to any retinal degeneration.^27,28^ Current research has not illuminated the extent to which cone opsins and cone opsin signaling direct outer retinal circuitry, but cone opsin gene regulatory sequences are active in mouse bipolar cells.^29^ In another mouse model lacking a cone outer segment ion channel, cones similarly survived with disrupted outer segments,^30^ and bipolar cells that would have otherwise synapsed with cones formed ectopic connections with rods.^31^

Gene therapy has been successfully used to restore opsin expression in mice in recent years. Zhang et al. used an AAV viral vector delivery method to restore M cone function in MKO mice.^31^ Ma et al. found that delivery of human L opsin genes via an AAV vector into their DKO mice before the age of two months rescued cone function for at least eight months post-treatment.^10^ Without this injection, however, their mice experienced significant retinal degeneration by three months. The mouse model presented here does not undergo degeneration for up to 12 months, making it a strong candidate for future gene therapy experiments targeted at restoring opsin production in cones. The total lack of background opsin expression in this model will allow for unambiguous and sensitive ERG recordings to detect effects of gene therapy interventions.

The viable and opsin-less cone population in this mouse line models the surviving but nonfunctional cones present in some cases of BCM. We demonstrated that these cells survive in a mouse retina in the absence of opsin, which supports the future use of this model as a gene therapy platform for the development of a BCM treatment. The viable cones will allow for research on the effects of viral delivery of an opsin gene. Further, this model will elucidate potential roles of cone opsins in the formation and maintenance of cone retinal circuits.

## Methods

### Genomic Editing

A Cas9-encoding plasmid and 4 single-guide RNAs (Table 1) flanking the gene to be knocked out were electroporated into wildtype Crb1 murine embryonic stem cells of C57BL/6NTac:129S6/SvEvTac F1 background, and candidate clones were transiently selected using 1.5 ug/mL puromycin. Positives clones were obtained by Taqman Velocigene Technology.

### Animals

Experiments with animals were done according to ARVO guidelines and under the supervision of the University of Washington Institutional Animal Care and Use Committee. DKO mice on the C57BL/6J background were created using gene editing techniques to completely remove both cone opsin genes and acquired from Regeneron. They were compared to MKO mice generated using the same methods as the DKO mice, C57BL/6J WT control mice and AIPL1^−/−^ C57BL/6J degeneration model mice. DKO retinas were studied at 4 months (4 m/o; n = 8) and 12 months (12 m/o; n = 7), MKO retinas were studied at 4 m/o (n = 3) and 12 m/o (n = 6), WT retinas were studied at 4 m/o (n = 3) and 12 m/o (n = 4), and AIPL1^−/−^ retinas were studied at 2 weeks (2 w/o; n = 1 homozygote).

### Electroretinogram Recordings

DKO mice were anesthetized using ketamine and xylazine, then underwent electroretinograms (ERGs) as described previously^32^ on a custom-built system. An OL-490 (Gooch and Housego) spectrally tunable light source’s output was integrated into a homogeneous, full-field (~ 120 degrees) Maxwellian View optical pathway and delivered to the mouse eye. Both photopic and mesopic conditions were collected via an ON-OFF paradigm with a base frequency of 1.1 Hz, with the spectrum tuned to 559 nm peak and 20 nm bandwidth at 100% of maximum (353 μW at the pupil plane) for cone ERGs, and the spectrum tuned to 500 nm peak at 20 nm bandwidth at 0.125% of maximum (34.25 μW at the pupil plane) for rod ERGs.

### Eye Processing

Mice were euthanized by administration of an intraperitoneal injection of Beuthanasia. Once confirmed dead, their eyes were dried with a tissue and marked on the nasal side using a black permanent marker for later orientation. Using forceps and dissecting scissors, the upper and lower eyelids were removed from each eye. On each side, the eye was pulled away from the skull by inserting curved forceps behind the eyeball and pushing it forward. Scissors were placed on the skull side of the forceps and used to cut through the connective and nervous tissue holding the eye to the skull. Once separated, the eye was placed immediately in 4% paraformaldehyde diluted in phosphate buffered saline (PBS, pH 7.4), and the process was repeated for the other eye.

After both eyes had been removed, each eye was dissected to remove the anterior portion of the eyes. Under a microscope, the corneas were pierced using a 25G needle to allow fixative to enter the eyes. Then, using dissecting scissors and forceps, the anterior portion of the eyes were removed, including the cornea, vitreous, and lens, leaving the eye cup. Using the ink mark for reference, a small, vertical incision was made on the nasal side of the retina. These tissues were nutated for an additional 2 hours in 4% paraformaldehyde at room temperature (RT) to ensure fixation.

The eye cups were cryoprotected using a series of sucrose solutions (10% and 30%). They were then embedded in OCT (Optimal Cutting Temperature, a water-soluble embedding medium for cryosectioning) in a superior to inferior orientation determined by the nasal side incision. These blocks were frozen and then sectioned into 20μm cryosections. Cryosections were placed on glass slides (Fisher Cat #22–035813) and stored at −20°C until staining.

### Immunostaining

The sections were stained using immunohistochemistry (IHC) techniques. Barriers were drawn around each staining well using an Immedge pen (Vector Labs #H-4000). Sections were first washed with PBS for 10 minutes, then blocked in blocking buffer (5% normal donkey serum, 1% bovine serum albumin, 1% Triton X-100 in PBS, pH 7.4) for one hour at RT. They were then incubated in primary antibodies and/or rhodamine-conjugated peanut agglutinin (PNA) diluted in blocking buffer for 24 hours at RT. Following this, the sections were washed in PBS again (three rounds of washing, each 10 minutes long) and the secondary antibodies conjugated to AlexaFluor dyes, and the nuclear counterstain Hoechst 33258 (Invitrogen #H3570, 1:1,000) diluted in blocking buffer were applied for a 2 hour incubation at RT. They were washed in PBS a third time (three rounds of washing, each 10 minutes long), then mounted with VectaShield (Vector Labs #H-1700) and a glass coverslip. Table 2 lists antibodies and stains used in this study.

### Imaging and Data Processing

Sections were imaged with a Leica SP8 confocal microscope using 20X and 63X oil objectives and LAS-X Software. The images were deconvolved using Leica Lightning, then processed and maximum intensity Z-projected using ImageJ. Images presented are maximum intensity projections over the full 20μm Z depth of each section.

Quantification of photoreceptor numbers and density was performed using ImageJ and Microsoft Excel. A line was added manually using ImageJ along interface between the photoreceptor layer and the retinal pigment epithelium (RPE) in sections stained with the cone arrestin (CArr) antibody and/or PNA; these sections were digitally straightened along this line. The point tool was then used to select each visible cone along the length of the section, and measurements were taken from 1–2 sections per animal in the inferior – superior plane across the central plane containing optic nerve. Data on the number and location of cones relative to the optic nerve head was imported from ImageJ to Excel. Excel was used to generate histograms based on these data, with a bin width of 100 μm. Bar graphs represent average cone densities in inferior and superior retinal regions of each type of animal. The RealStatistics resource pack for Excel was used for statistical testing.

### Serial Block-face Scanning Electron Microscopy

Mouse eyes were removed as described above and placed immediately into fixative (4% glutaraldehyde in 0.1 M sodium cacodylate buffer, pH 7.2). Posterior eyecups were dissected, quartered, fixed overnight at RT, then stored overnight at 4°C. Samples were washed 4 times in sodium cacodylate buffer, post-fixed in osmium ferrocyanide (2% osmium tetroxide/3% potassium ferrocyanide in buffer) for 1 h on ice, washed, incubated in 1% thiocarbohydrazide for 20 min, and washed again. After incubation in 2% osmium tetroxide for 30 min at RT, samples were washed and en bloc stained with 1% aqueous uranyl acetate overnight at 4°C. Samples were finally washed and en bloc stained with Walton’s lead aspartate for 30 min at 60°C, dehydrated in a graded ethanol series, and embedded in Durcupan resin. Unless stated otherwise, five washes with water were used for all wash steps. Resin blocks were mounted in a Zeiss Sigma VP scanning electron microscope fitted with a Gatan 3View2XP ultramicrotome apparatus. Serial sections were cut at 60 nm thickness and imaged with 6 nm pixel size. 3-D volumes of the photoreceptor layer were aligned, stitched,^33^ and annotated using the TrakEM2 plugin^34^ for ImageJ.

## Supplementary Material

Supplementary Files

This is a list of supplementary files associated with this preprint. Click to download.

- Picture6.jpg
- Picture7.jpg

Tables are available in the Supplementary Files section.

## Data Availability

All data supporting the findings of this study are available within the paper.
